# A recommendation for the management of lesions of unknown malignancy in multiple primary malignant neoplasm patients: A case report

**DOI:** 10.3892/ol.2014.2601

**Published:** 2014-10-10

**Authors:** XIANGYU XIA, KAIJUN CUI

**Affiliations:** 1 Cancer Center, West China Hospital, West China Medical School, Chengdu, Sichuan 610041, P.R. China; 2 Department of Cardiology, West China Hospital, West China Medical School, Chengdu, Sichuan 610041, P.R. China; 3 State Key Laboratory of Biotherapy, Sichuan University, Chengdu, Sichuan 610041, P.R. China

**Keywords:** carcinoma, cancer, multiple, management, hepatocellular, rectum, bladder

## Abstract

In numerous patients with multiple primary malignant neoplasms, it is difficult to determine whether the mass is benign or malignant, and the method to treat these lesions is controversial. For patients with a history of cancer, a point of high-risk for the development of a second primary tumor occurs following a 10-year gap. To the best of our knowledge, to date, there has been no large clinical trial to observe the appropriate method to manage the lesions in patients with multiple primary malignant neoplasms. The present study reports the case of a patient who was initially diagnosed with rectal cancer, treated with Dixon’s rectectomy and post-operative chemotherapy. The patient’s disease was evaluated as complete response following these treatments. However, the patient then presented with bladder cancer and underwent transurethral resection of the bladder tumor, again achieving a complete response. The patient more recently presented with hepatocellular carcinoma, which developed from an unexplained mass in the liver. The patient underwent partial liver resection and to date, has achieved a complete response. The management of masses of unknown malignancy is also discussed. The current case provides useful insight for future research in this field.

## Introduction

The definition of multiple primary malignant neoplasms (MPMNs) was determined by Warren and Gates in 1932 (1) and since then, an increasing number of MPMNs have been diagnosed and reported. MPMN patients usually undergo surgery or receive chemotherapy for treatment (2,3). Additionally, with the increasing lifespan of humans, the prevalence of MPMN has increased (4). Prior to diagnosis, a number of patients with this condition have masses of unknown malignancy, and the method to treat these lesions is controversial. One study has proposed the observation of such lesions instead of biopsy (5). The present study reports the case of a patient who was diagnosed with rectal and bladder cancer, and later presented with hepatocellular carcinoma, which developed from an unexplained mass in the liver during surveillance following surgery, and provides a promising method of treatment. This case provides valuable insight for further research in this field. The patient provided written informed consent.

## Case report

**Figure 1:**
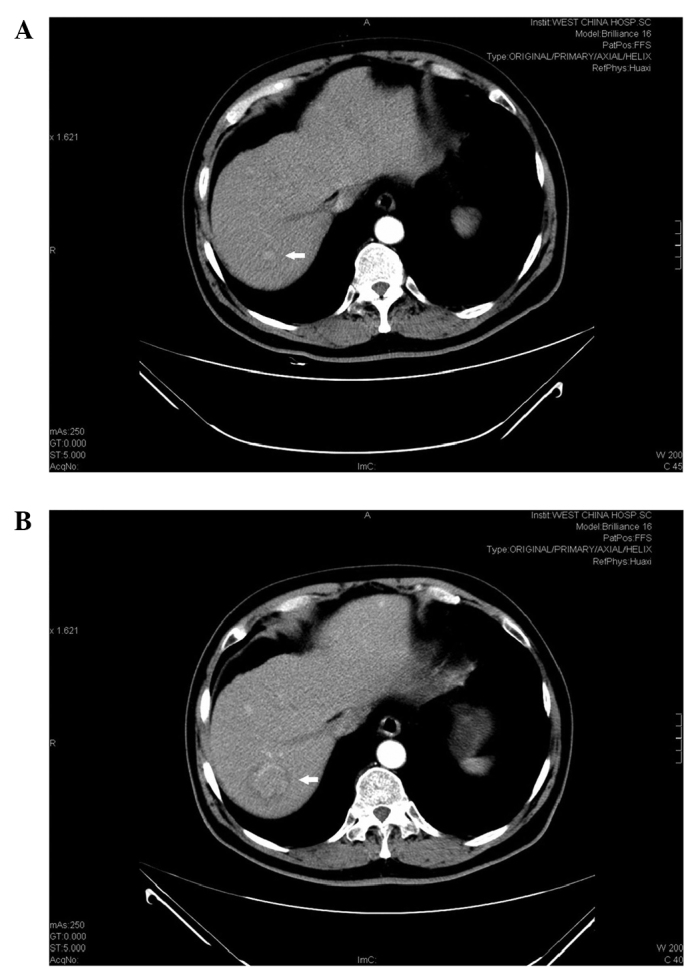
(A) The 0.9-cm tissue mass of unknown malignancy: A contrast computed tomography scan on December 3, 2010, showed a mildly-enhanced lesion in the right lobe of the liver when it was discovered for the first time. (B) The mass of 3 cm in diameter was confirmed as hepatic carcinoma by computed tomography on December 20, 2011.

A 62-year-old male presented to the West China Hospital of Sichuan University (Chengdu, China) due to blood in the feces and weight loss that had been occurring for approximately one month. A proctoscopy indicated a rectal adenoma. At day seven post-admission, the patient underwent Dixon’s rectectomy. During the surgery, a 5×4-cm neoplasm was observed in the rectum and diagnosed as hepatic cirrhosis. Following the surgery, the microscopic examination confirmed the neoplasm to be a rectal adenoma. The patient received four cycles of post-operative chemotherapy composed of a 5-Fu infusion (250 mg/day from days one to three). However, due to intolera- ble rashes, the treatment was changed to 200 mg tegafur (three times per day on days four to 10) for the first cycle, while for the following three cycles the tegafur was administered at the same dose and frequency but for 10 days. The patient also received 41 doses of T-cell therapy (20 ml infusion ever two or three days). There were no complications in the procedure. Additionally, a hepatitis B virus (HBV) test showed that the patient was positive for the HB surface, envelope and core antigens. The HBV DNA content was 2.85×10^6^/ml. The patient received glutathione (1,200 mg/day during hospitalization) and bifendate (15 mg, three times per day until the HBV-DNA levels had ret- urned to normal) as liver treatment. The patient achieved a complete response following these treatments. Approximately seven months later, the patient required hospitalization due to the chief complaint of painless gross hematuria persisting for 1 week. Ultrasonography showed a mass in the urinary bladder, which did not move with the change of body position. At day four post-admission, the patient received a transurethral resection of the bladder tumor. Following the surgery, bladder instillation was performed for treatment with doxorubicin. The post-operative biopsy of the neoplasm, with hematoxylin and eosin staining revealed that the pathological type was a bladder transitional cell carcinoma, following assessment by a pathologist from the West China Hospital. Eight years after the second surgery, a mass of 0.9 cm in diameter was discovered in the liver by contrast computed tomography (Fig. 1A). Approximately one year later, this mass developed into hepatic cancer in the right posterior lobe of the upper section of the liver (Fig. 1B). Subsequently, the patient received a partial liver resection, the hepatic mass was stained with hematoxylin and eosin and was confirmed as hepatocellular carcinoma by pathological analysis, by a pathologist from the West China Hospital. After this last surgery, the patient recovered well and was disease-free with an Eastern Cooperative Oncology Group score of 1.

## Discussion

The description of MPMN provided by Warren and Gates in 1932 is the generally accepted diagnostic standard for this disease: Each of the tumors must have a definite element of malignancy; each one must be distinct; and the probability of one being a metastasis of other tumors must be excluded (1). The concept of metachronous cancers is described as two or more tumors detected six months following the primary tumor (6). According to the aforementioned criteria, the present patient suffered from metachronous MPMN. The prevalence of MPMN is not high, varying between 0.341 and 5.464% (7–12). It has been reported that the extended life span of humans and the continuous persistence of carcinogens are significant factors for the tumorigenesis of MPMN. A previous study has shown that, among the patients with brain malignant tumors, the incidence of MPMN has two peaks. The first is in the third decade of life, and the second is after 50 years of age (4). A point of high-risk for the development of a second primary tumor occurs following a 10-year gap from the first (13). Although Evans *et al* (14) concluded that the risk of developing a subsequent cancer in older patients is lower than expected, the study did indicate that this may result from the incomplete design of the experiment and the lack of data.

More significantly, the present study observed a 0.9-cm mass in the right lobe of the liver. When considering the histological type prior to biopsy, three possibilities persisted: A benign lesion, another primary cancer or a metastasis of a resected tumor (1). Generally, for single abdominal masses of <1 cm, they can be kept under surveillance for dynamic observations (5). However, the mass in the present case developed into hepatocellular carcinoma 1 year later. Hence, to monitor patients with an oncological history, particularly of colorectal cancer, and an unexplained mass in the liver, it is necessary to screen the α-fetoprotein (AFP) level. However, the level of AFP in certain patients with HCC and those of biliary tract cancers does not become elevated (15,16). Due to the unraised AFP level, a biopsy should be taken. If malignancy is confirmed, surgery should be performed as soon as possible to minimize the risk of invasion and metastasis of the carcinoma.

In conclusion, older patients have a predisposition to developing MPMN. Active biopsies for suspected hepatic disease found upon imaging in patients with a history of cancer is a prospective method for the early diagnosis of emerging carcinoma.
